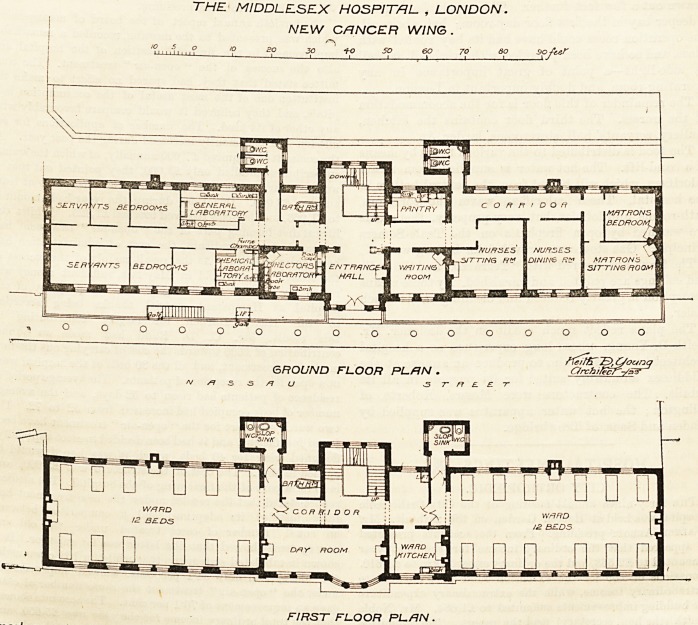# Hospital Construction

**Published:** 1900-04-07

**Authors:** 


### The Institutional Workshop.

### HOSPITAL CONSTRUCTION.

### THE NEW CANCER WARDS AT THE MIDDLESEX HOSPITAL.

The erection of blocks at our large general hospitals has at least one good result. It must infallibly diminish the number of small special hospitals which have lately sprung up in so many of the London districts. We print with this the ground and first floor plans of the

THE MIDDLESEX HOSPITAL, LONDON.
NEW CANCER WING.

FIRST FLOOR PLAN.

most recent addition of these special blocks to the Middlesex Hospital. It is in every respect a complete self-contained hospital, except that it is not provided with either a separate laundry or with its own mortuary.

The basement is utilised chiefly as a storeroom for linen, hardware, and such things, but one room is fitted up as a laboratory for the preparation of various media used in research work, and another is used as workshop for the electrician. As an instance of the extent to which bicycles are now used by the staffs of hospitals, it should be noted that the architect has not only set apart a special room for these machines, but has constructed a special staircase with sloping track for access to the front pavement.

It will be seen that the ground floor has a fine entrance hall, and that the main staircase is placed immediately behind this. The latter has in its well a passenger lift worked by electricity. On the right of the hall is the matron's office or waiting-room; then follow the nurses' sitting-room and dining-room, and the matron's private apartments.

On this floor three rooms are set apart as laboratories, so that it is evident that scientific and original research will play an important part in the work of this annexe. There are seven bedrooms for servants. The closet blocks are properly cut off from the main by cross-ventilated passages. A bath is close to one of these blocks, where it is in its proper position; but we could wish that the pantry had not been so near to the other sanitary blocks.

The first floor is admirably arranged. The centre contains a day-room with a circular bay, a ward kitchen, a bath-room, and the sanitary blocks, which, as we have already stated, are correctly planned. There are two wards, each for twelve beds. These rooms occupy, so to speak, the wings of the block, and each is about 25 ft. wide by 52 ft. long. The height is not stated, but placing it at 12 ft. we should have 1,300 cubic feet per bed.

On the second floor there is only one ward, with its ward kitchen. The space occupied by the day-room on the first floor is here divided into a day-room and an operation-room. Presumably the latter is the part having the circular bay. Here an improvement might have been made. The projecting part of the porch, and, of course, the basement under it, should have been drawn out a few feet further. This would have given a deeper bay in the first floor day-room; but, above all, the operation-room could have had its bay roofed with glass, and so have been provided with overhead as well as side-light—a point of great importance in any operating-room, and doubly important in London.

The remainder of this floor is for the accommodation of the nurses. The third floor contains the kitchen, scullery, servants' hall, storerooms, larder, &c.

The food is distributed to the various floors by means of a hand-lift. The hot water is supplied from steam calorifiers, which will be fed from the central boilers in the hospital. These calorifiers warm the corridors, bath-rooms, and lobbies, but, very properly, the wards are warmed by open fireplaces on the Teale-Somers principle. Gas-stoves are provided for boiling water.

The floors are covered with "Terrazzo," and the walls and ceilings are painted and varnished. By these means absorption is greatly reduced and cleaning made much easier.

The plans reflect much credit on the designer, Mr. Keith D. Young, whose long experience as a hospital architect has enabled him to produce an annexe at the Middlesex admirably suited for its purpose in all its details. The contractors were Messrs. Roberts, of Islington; the hot water apparatus was supplied by Haden and Sons, of Trowbridge.

## Figures and Tables

**Figure f1:**